# Clinical outcomes of patients diagnosed with cancer of unknown primary or malignancy of undefined primary origin who were referred to a regional cancer center

**DOI:** 10.1007/s10147-023-02316-y

**Published:** 2023-03-10

**Authors:** Masashi Ando, Kazunori Honda, Waki Hosoda, Yuki Matsubara, Ryosuke Kumanishi, Taiko Nakazawa, Takatsugu Ogata, Akinobu Nakata, Hiroyuki Kodama, Toshiki Masuishi, Yukiya Narita, Hiroya Taniguchi, Shigenori Kadowaki, Kei Muro

**Affiliations:** 1 Department of Clinical Oncology, Aichi Cancer Center Hospital, 1-1 Kanokoden, Chikusa-ku, Nagoya, Aichi Japan; 2 Pathology and Molecular Diagnostics, Aichi Cancer Center Hospital, 1-1 Kanokoden, Chikusa-ku, Nagoya, Aichi Japan

**Keywords:** Cancer of unknown primary, Unfavorable risk, Immunohistochemistry, Predicted primary site, Site-specific therapy

## Abstract

**Background:**

A regional cancer hospital has been identified to be crucial in the management of malignancies of undefined primary origin (MUO) and cancer of unknown primary (CUP). This hospital primarily consists of oncologists with expertise in CUP, pathologists, and interventional radiologists. Early consultation or referral of MUO and CUP to a cancer hospital is deemed important.

**Methods:**

This study retrospectively collected and analyzed the clinical, pathological, and outcome data of all patients (*n* = 407) referred to the Aichi Cancer Center Hospital (ACCH) in Japan over an 8-year period.

**Results:**

In total, 30% of patients were referred for a second opinion. Among 285 patients, 13% had non-neoplastic disease or confirmed primary site and 76% had confirmed CUP (cCUP), with 29% of cCUP being identified as favorable risk. In 155 patients with unfavorable-risk CUP, 73% had primary sites predicted by immunohistochemistry (IHC) and distribution of metastatic sites, whereas 66% of them received site-specific therapies based on the predicted primary sites. The median overall survival (OS) was found to be poor in patients with MUO (1 month) and provisional CUP (6 months). In addition, the median OS of 206 patients with cCUP treated at the ACCH was 16 months (favorable risk, 27 months; unfavorable risk, 12 months). No significant difference was noted in OS between patients with non-predictable and predictable primary-sites (13 vs 12 months, *p* = 0.411).

**Conclusion:**

The outcome of patients with unfavorable-risk CUP remains to be poor. Site-specific therapy based on IHC is not recommended for all patients with unfavorable-risk CUP.

**Supplementary Information:**

The online version contains supplementary material available at 10.1007/s10147-023-02316-y.

## Introduction

Cancer of unknown primary (CUP) accounts for 3–5% of all adult malignancies, with recent studies reporting that the incidence of CUP had fallen to around 2% due to improved diagnostic methods [1–3]. CUP is a heterogeneous group of metastasizing tumors for which routine diagnostic workup fails to identify the site of origin at the time of diagnosis [4].

Patients with metastatic malignancy identified on a limited number of clinical or radiological examinations, but no obvious primary-site, are often referred to as having “malignancy of undefined primary origin (MUO)” [5]. Patients with CUP can be further classified as having provisional CUP (pCUP, metastatic epithelial or neuroendocrine malignancy identified on histology or cytology, with no primary site detected despite a selected initial screen of investigations, before specialist review and possible further specialized investigations) or confirmed CUP (cCUP, patients with pCUP who underwent specialist review and further specialized investigations as appropriate) [5]. These definitions (MUO, pCUP, and cCUP) can vary depending on the extent of investigation and whether patients have been reviewed by oncologists with CUP expertise, and patients’ general condition at the time of visit [5].

Approximately, 15–20% of patients with CUP belong to clinicopathological subsets with a more favorable prognosis, chemosensitivity and long-term disease control with specific therapies (favorable-risk CUP) [4]. However, the remaining 80% were categorized as unfavorable-risk CUP without specific therapies [6].

The Aichi Cancer Center Hospital (ACCH) has played an important role in the management of MUO and CUP, in the Tokai region of eastern Japan; this hospital is supported by oncologists with expertise in CUP, pathologists, and interventional radiologists [7]. In this study, we analyzed the clinical data and assessed the survival of patients with MUO or CUP who were referred to the ACCH over a period of 8 years.

## Patients and methods

### Study population

In this study, all patients who were referred to the ACCH between July 2012 and December 2020 and diagnosed with MUO or pCUP following initial examinations were retrospectively reviewed. All cases were discussed in the Department of Clinical Oncology at the ACCH, including radiological and pathological reviews. Patients who did not receive biopsy or additional examinations due to frailty, clinical deterioration, or patient refusal were diagnosed with MUO, whereas those who were clinically and radiologically reviewed at the ACCH, and whose biopsy revealed non-malignant findings, were classified as having “non-malignant disease.” In addition, patients who were reviewed at the ACCH and whose clinical, radiological, endoscopic, and histological patterns suggested a specific primary cancer were defined as having “primary cancer identified.” Before pathological reviews at the ACCH, patients with histological evidence of malignancy who had completed thorough examinations without identifying the primary site were classified as pCUP. After clinical and pathological reviews at the ACCH, patients with pCUP were considered cCUP. Comprehensive examinations included the following: medical history and physical examination (including otolaryngology examination, urology examination in men, and gynecological examination in women); laboratory tests including serum tumor markers such as cancer antigen 125 in women, prostate-specific antigen in men, human chorionic gonadotropin, and alpha-fetoprotein; radiological investigations (including computed tomographic scan or positron emission tomography of the thorax, abdomen and pelvis, mammography in women); gastrointestinal endoscopy (in cases with any digestive symptoms, positive fecal occult blood, or abdominal lesions); and urinary cytology. Additional diagnostic tests were performed if clinically indicated. Examinations were done if clinically indicated. All biopsy or an open biopsy, while those with only ascites or pleural effusion, had cytology and cell block methods used for pathological diagnosis.

According to the European Society for Medical Oncology (ESMO) guideline (2015) [4], patients with cCUP were classified as having favorable-risk CUP if they had one of the following; isolated axillary nodal metastases of adenocarcinoma in women, peritoneal adenocarcinomatosis (Peri ADC) of a serous papillary histological type in females, osteoblastic bone metastases of adenocarcinoma with positive immunohistochemical (IHC) staining of prostate-specific antigen (PSA) or elevated serum PSA in males, liver or peritoneal metastases of adenocarcinoma with a colorectal cancer immunoprofile (CK7/CK20− /+ and CDX2 ), well or poorly differentiated neuroendocrine tumor of unknown primary, squamous cell carcinoma in cervical lymph nodes, and a single metastatic lesion of unknown primary. All patients who did not fall into one of the favorable-risk subgroups were considered to have unfavorable-risk CUP.

### Pathological assessment

**Fig. 1:**
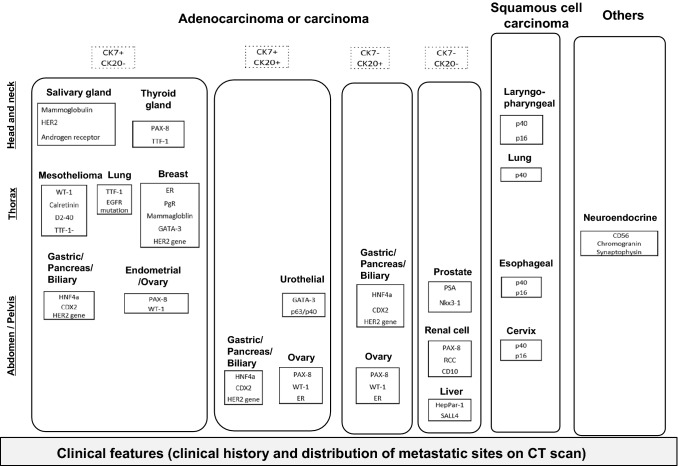
An IHC panel for predicting primary sites (modified version of the previous study by Hasegawa H et al. [7]). The initial assessment was based on morphological features, such as adenocarcinoma, squamous cell carcinoma, and others. Furthermore, primary sites were speculated based on confirmatory immunohistochemistry using cancer-specific antibodies, taking into account of clinical history and the distribution of metastatic sites. *IHC* immunohistochemistry

At the ACCH, biopsy specimens were reviewed by three pathologists, and the most likely primary site or sites of the carcinoma were identified by integrating morphology with IHC for organ-restricted markers. A panel of well-performed and well-interpreted antibodies of organ-restricted markers was used for patients diagnosed with a malignant epithelial tumor, including those of lineage-specific transcription factors such as thyroid transcription factor-1 (TTF-1), PAX-8, GATA-3, hepatocyte nuclear factor 4 alfa (HNF-4 alfa), CDX2, SATB2, NKX3.1, SALL4, and other organ-specific markers such as estrogen receptor, progesterone receptor, androgen receptor, calretinin, mesothelin, D2-40, mammaglobin, gross cystic disease fluid protein-15 (GCDFP-15), HER2, CD10, PSA, p16, p40, chromogranin, synaptophysin, CD56, Melan-A, HMB-45, S-100, and PLAP. Cytokeratins (CK7 and CK20), which are known to have a unique distribution in normal epithelium and carcinomas, were used to identify adenocarcinomas [8, 9]. In addition to IHC examinations, genetic analyses for *HER2* gene amplification or *RAS*, *BRAF*, and *EGFR* mutations were performed if they were informative for further differential diagnosis [10–14]. Figure 1 depicts the IHC panel used to predict primary sites, which is a modified version of the previous study [8]. In brief, morphological features such as adenocarcinoma, poorly differentiated carcinoma, squamous cell carcinoma, neuroendocrine tumors (well or poorly differentiated), and others were used for initial assessment. Following that, the results of an IHC panel using organ-restricted antibodies, distribution of metastatic sites, and clinical history were used to identify the most likely primary sites. Predicted primary sites were determined as follows: breast/skin appendage/salivary gland cancer (BSSC) profile, adenocarcinoma, GATA3+ , GCDFP-15+ or −, mammaglobin+ or −, and not applicable to isolated axillary nodal metastases of adenocarcinoma in women of favorable-risk subtype; non-small cell lung cancer (NSCLC) profile, TTF-1+ or −, CK7+ , CK20 − , EGFR mutation+ or − , excluded germ cell tumor, and mainly of mediastinal lymph nodes; gastric/pancreatic/bile duct cancer (GPBC) profile, adenocarcinoma, CK7/20± or ± or + / + , HNF-4alpha+ , and mainly of intra-abdominal lesions; urinary tract cancer (UTC) profile, GATA3+ , and excluded breast cancer; renal cell cancer (RCC) profile [15]^)^, CK7+ or −, clear cell or papillary, PAX8 + , and excluded thyroid and ovarian cancer; ovarian/endometrial cancer (OEC) profile, women, adenocarcinoma, PAX8 + , excluded renal cell and thyroid cancer, calretinin−, mainly of intra-abdominal lesions, and not applicable to peritoneal adenocarcinomatosis of a serous papillary histological type in women of favorable-risk subtype; uterine cervical cancer (UtCC) profile [16]^)^, women, squamous cell, p16+ , and mainly of intrapelvic lymph nodes; melanoma of unknown primary, cytokeratin−, LCA−, S-100+ , HMB-45+ , and Melan-A+ .

### Treatment

If clinically indicated, patients had received palliative radiotherapy for bone, or brain metastasis and/or surgery such as lymph node dissection and vertebral fixations for bone metastases. In addition, patients with Eastern Cooperative Oncology Group (ECOG) performance status (PS) 0–2 and adequate organ function were treated with systemic therapy as first-line treatment at the ACCH. Patients with favorable-risk CUP were also treated in the same way as patients with known primary cancers for metastatic disease [4]. Furthermore, patients with unfavorable-risk CUP were given site-specific therapy for each predicted cancer profile, while those with no predictable cancer profile were given empiric chemotherapy including platinum combination regimens.

### Statistical analysis

Overall survival (OS) was defined as the time between the first visit to the ACCH and death from any cause or the last follow-up. OS was estimated using the Kaplan–Meier method and compared using the log-rank test. Time to treatment failure (TTF) was defined as the time between the start of first-line therapy and the start of the following therapy, death from any cause, or last follow-up. The follow-up time was defined as the time between the first visit and death from any cause or the last follow-up. Multivariate analyses were performed using the Cox proportional hazards model, including three prognostic significant factors in the univariate analysis for OS, such as ECOG performance status, number of metastatic sites, and presence of liver metastasis. All statistical analyses were performed using EZR, R 4.0.3 (R Foundation for Statistical Computing, Vienna, Austria).

This study was approved by the Institutional Review Board of the Aichi Cancer Center Hospital (approval no. 2021-0-150).

## Results

### Patient characteristics

**Fig. 2:**
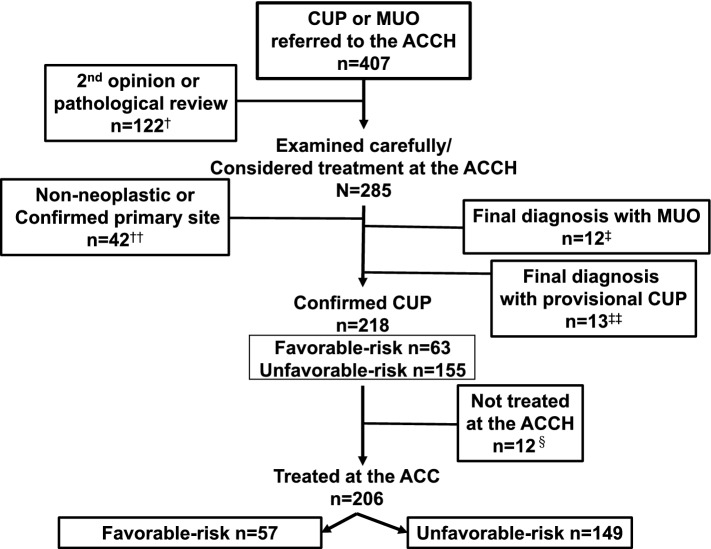
Study profile between July 2012 and December 2020. 407 patients with cancer of unknown primary (CUP) or metastatic malignant disease of unknown primary origin (MUO) were referred to the Aichi Cancer Center Hospital (ACCH). ^†^Patient`s characteristics; male 59%, median age 65 years, range: 29–91 years, and eight patients were referred for only pathological review. ^††^One patient was diagnosed with hemangioma of liver, and three with non-neoplastic disease. ^‡^Patients were finally diagnosed with MUO after failing to undergo thorough examinations, and they had only received the best supportive care (*n* = 9), or radiotherapy (*n* = 3). ^‡‡^ Patients diagnosed with provisional CUP at the referrer hospitals did not obtain additional examinations to confirm CUP due to poor general condition or complications or comorbidities, and they had only received the best supportive care (*n* = 7), radiotherapy (*n* = 4), and surgery for bone metastasis (*n* = 2). ^§^Twelve patients received treatments at the referrer hospitals

Over an 8.5-year period, 407 patients diagnosed with MUO or CUP were referred to the ACCH (Fig. 2). Among them, 122 patients (30%) were referred to the ACCH for a second opinion or pathological review. Meanwhile, the remaining 285 patients underwent clinical examinations and pathological reviews at the ACCH, with 32 patients (8%) having a history of malignant neoplasm. After thorough examinations and pathological reviews at the ACCH, 38 patients (13%) had primary sites identified (Table 1), while 1 patient had hemangioma of the liver and 3 had non-neoplastic disease: 1 with bone marrow hyperplasia with a solitary lesion of the cranial bone and 2 with peritoneal tuberculosis, which resembled peritoneal metastases.

**Table 1:** Patient characteristics

Final diagnosis at the ACCH	MUO (*n* = 12)	Provisional CUP (*n* = 13)	Confirmed CUP (*n* = 218)	Primary site identified (*n* = 38)
	*n*	*n*	*n*	*n*
Age median (range)	61 (42–80)	73 (54–88)	67 (28– 83)	65 (22 – 86)
Sex				
Male/female	7/5	8/5	104/114	20/18
ECOG performance status* (range)	3 (1–4)	2 (1–4)	1 (0–4)	1 (0 – 4)
Number of metastatic sites				
1	5	6	121	20
2	5	5	57	11
3	1	0	24	5
4 +	1	2	16	2
Sites of disease				
Bone	5	5	55	11
Lung	1	3	20	9
Liver	3	1	34	3
Lymph node	4	8	122	23
Peritoneum	5	2	72	4
Biopsy				
Referrer hospital/ACCH				
Not done/done	0	3	45	9
Done/not done	9	9	107	10
Done/done	1	0	45	19
Not done/not done	2 (cytology)	1 (cytology)	21 (cytology)	0
Histopathology				Primary site
Adenocarcinoma	3	6	134	Lung 5
Carcinoma (P/D, U/D)	2	5	33	Urinary tract 5
Squamous cell carcinoma	2	2	31	Lymphoma 5
Neuroendocrine tumor/carcinoma	0	0	11	Ovary/fallopian tube 3
Others	4**	0	9**	Uterine body/cervix 3 Soft tissue sarcoma 3 Gastric 2 Colon/appendix 2 Prostate 2 Skin appendage 2
				Others*** 6

*ACCH* Aichi Cancer Center Hospital, *M/D* moderately differentiated, *MUO* metastatic malignant disease of unknown primary origin, *P/D* poorly differentiated, *U/D* undifferentiated

*At the time of the first visit to the ACCH

**Clear cell carcinoma 2, adenoasquamous carcinoma 1, melanoma 2, sarcomatoid carcinoma 2, papillary renal cell carcinoma 1, myoepithelial carcinoma1

***Pancreas 1, gall bladder 1, thyroid 1, breast 1, extramammary Paget’s disease 1, malignant peritoneal mesothelioma 1

Twelve patients were finally diagnosed with MUO at the ACCH after failing to undergo thorough examinations due to poor condition, comorbidities, or the patients’ wishes.

In addition, due to poor general condition, complications, or comorbidities, 13 patients diagnosed with pCUP at the referrer hospitals did not obtain any additional examinations to confirm CUP, and the diagnosis of pCUP remained at the ACCH. As a result, 218 of 285 patients (76%) were eventually diagnosed with cCUP (Table 1).

#### Predicted primary-sites based on immunohistochemistry

All patients with cCUP (*n* = 218) received pathological reviews, while biopsies and IHC examinations were performed at a referrer hospital and the ACCH on only 45 patients (21%), due to lacking materials for additional IHC examinations (Table 1). In addition, cytology and cell block methods were used on 21 patients (10%) who had ascites and peritoneal dissemination and were all diagnosed with Peri ADC. The median number of IHC examinations performed on each patient was six (range 2–11). Genetic analyses were performed in 30% of patients with cCUP. Moreover, 11 patients (17%) were in the colorectal cancer profile, with RAS and BRAF gene mutations detected in 2 and 1 of them, respectively. All four patients with a single metastatic lesion were determined to have an inguinal lymph node, while three had squamous cell carcinoma.

Of the 155 patients with unfavorable-risk subgroups, 73% were predicted for primary sites by the IHC panel (Fig. 1, Table 2). In 114 patients with a predicted primary site, the GPBC profile was more frequent (41%). The remaining 41 patients (26%) had unpredictable primary-sites (Appendix Table 1).

**Table 2:** Confirmed cancer of unknown primary, risk subgroups, and predicted primary sites of unfavorable risk using an IHC panel (Fig. 1)

Confirmed cancer of unknown primary	218
Risk subgroup	*n* (%)
Favorable-risk subgroup (accounting for 29% of confirmed cancer of unknown primary)	63
Isolated axillary nodal metastases of adenocarcinoma in women	3 (5)
Peritoneal adenocarcinomatosis of a serous papillary histological type in women	29 (46)
Liver or peritoneal metastases of adenocarcinoma with colorectal cancer profile	11 (17)
Well-differentiated neuroendocrine tumor of unknown primary	1 (2)
Poorly differentiated neuroendocrine carcinoma of unknown primary	10 (16)
Squamous cell carcinoma in cervical lymph nodes	5 (8)
Single metastatic lesion of unknown primary	4 (6)
Unfavorable-risk subgroup (accounting for 71% of confirmed cancer of unknown primary)	155
Non-predicted primary site	41 (26)
Breast/skin appendage/salivary gland cancer profile	14 (9)
Non-small cell lung cancer profile	8 (5)
Gastric/pancreatic/bile duct cancer profile	64 (41)
Urinary tract cancer profile	9 (6)
Renal cell cancer profile	4 (3)
Ovarian/endometrial cancer profile	6 (4)
Uterus cervical cancer profile	7 (5)
Melanoma of unknown primary	2 (1)

#### Treatment

Twelve patients with cCUP were treated at their referrer hospitals, with 6 of them having favorable-risk subtypes. On the other hand, 206 patients were treated at the ACCH (Appendix Table 2).

Moreover, 133 patients had received systemic therapy, and 54 of 58 patients with a GPBC profile received 5-fluorouracil (5-FU) or leucovorin plus oxaliplatin (mFOLFOX6) as first-line therapy (Appendix Table 3). In addition, 43 patients received platinum and taxane combination regimens. All four patients with an RCC profile received tyrosine-kinase inhibitor, while five received immune checkpoint inhibitor (ICI) as first-line therapy (two with melanoma of unknown primary profile, one with a GSPC profile with high microsatellite instability (MSI), and two with NSCLC and UtCC profiles with high programmed cell deth1-ligand1 (PD-L1) tumor proportion score (TPS). Among them, one patient with a GPBC profile and MSI-high has received pembrolizumab over 16 months. The median TTF at a first-line systemic therapy for the 133 patients was 4 months (95% confidence interval (CI): 3–5 months), with a trend of longer TTF in patients with BSSC, OEC, UtCC, and RCC profiles compared to those with non-predictable, GPBC, NSCLC, and UTC profiles (Table 2).

#### Overall survival

At a median follow-up time of 13 months (IQR: 6–25 months), the median OS for patients with MUO, pCUP, and cCUP at the ACCH was 0.75 month (95% CI: 0 – 3 months), 1 month (95% CI 1–12 months), and 16 months (95% CI 13–20 months), respectively (Appendix Figure). On the other hand, the median OS of 38 patients with identified primary sites was 24 months (95% CI: 6—not estimable), with no significant difference compared to those with cCUP (*p* = 0.497).

**Fig. 3:**
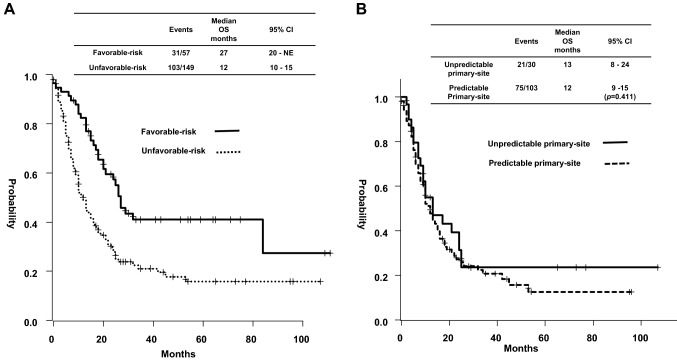
**A** Overall survival of 206 patients with cancer of unknown primary treated at the Aichi Cancer Center Hospital, according to risk subgroups. **B** Overall survival of 133 patients with unfavorable risk treated with systemic therapy, based on primary site prediction. *CI* confidence interval, *NE* not estimable, *OS* overall survival

The median OS of 57 patients with favorable-risk subgroups was 27 months, while that of patients with a Peri ADC profile, which accounted for 42% of these patients, was 84 months (Fig. 3A, Table 3). The median OS of 149 patients in the unfavorable-risk subgroup was 12 months. In 133 patients treated with systemic therapy, the median OS was 12 months (95% CI: 10–15 months). No significant difference was noted in the OS between patients with non-predictable (*n* = 30) and predictable primary sites (n = 103) with systemic therapy (13 vs. 12 months, respectively, *p* = 0.411, Fig. 3B). In 103 patients with predictable primary-sites, the median OS of patients with a GPBC profile was 8 months, whereas it was longer in patients with UtCC (*n* = 7) and RCC (*n* = 4) profiles (19 and 42 months, respectively, Table 3). A univariable and multivariable analysis for OS was performed using the baseline characteristics, including sex, age, ECOG PS, presence of bone or liver metastasis, and the number of metastatic sites at the start of treatment. In patients with unfavorable subgroups with systemic therapy. Liver metastasis, ECOG PS, and the number of metastatic sites were independent prognostic factors for OS (Appendix Table 4).

**Table 3:** Overall survival of 206 patients with cancer of unknown primary, treated at the Aichi Cancer Hospital

	Median overall survival months (95%CI)
All patients with cancer of unknown primary (*n* = 206)	16.0 (13–20)
Favorable-risk subgroups	
All (*n* = 57)^b^	27.0 (20-NE)
Isolated axillary nodal metastases of adenocarcinoma in women (*n* = 3)	75, 109, 151, alive
Peritoneal adenocarcinomatosis of a serous papillary histological type in women (*n* = 24)	84.0 (20-NE)
Liver or peritoneal metastases of adenocarcinoma with colorectal cancer profile (*n* = 9)^a^	25.0 (6-NE)
Well-differentiated neuroendocrine tumor of unknown primary (*n* = 1)	23
Poorly differentiated neuroendocrine carcinoma of unknown primary (*n* = 10)	12 (1–17)
Squamous cell carcinoma in cervical lymph nodes (*n* = 4)^a^	15 (15-NE)
Single metastatic lesion of unknown primary (*n* = 4)	27 + , 24,30, 71 alive
Unfavorable-risk subgroup	
All (*n* = 149)	12.0 (10–15)
Without systemic therapy (*n* = 16)^c^	6.0 (1-NE)
With systemic therapy (*n* = 133)	12.0 (10–15)
Non-predictable primary site (*n* = 30)	13.0 (8–25)
Predictable primary site (*n* = 103)	12.0 (9–15)
Breast/skin appendage/salivary gland cancer profile (*n* = 13)	NA (5-NE)
Non-small cell lung cancer profile (*n* = 8)	13.0 (2–23)
Gastric/pancreatic/bile duct cancer profile (*n* = 58)	8.0 (6–10)
Urinary tract cancer profile (*n* = 6)	9.5 (9-NE)
Renal cell cancer profile (*n* = 4)	42.0 (18-NE)
Ovarian/endometrial cancer profile (*n* = 5)	NA (3-NE)
Uterus cervical cancer profile (*n* = 7)	19.0 (9-NE)
Melanoma of unknown primary (*n* = 2)	12, 13

*CI* confidence interval, *NE* not estimable

^a^One patient received only the best supportive care

^b^51 patients received systemic therapy, and 4 received only local therapy (radiotherapy (*n* = 1) or surgical resection (*n* = 3))

^c^One had surgery for bone metastases, six had radiotherapy for brain or bone metastases, and nine received only the best supportive care

## Discussion

In this study, we present the retrospective case series and clinical outcomes of patients with MUO or CUP, who were referred to a regional cancer hospital in Japan. In the previous study of patients referred to a regional MUO/CUP service, 27% were diagnosed with MUO and had a poor prognosis, which is consistent with our study [18].

In this study, primary sites were identified in 13% of 285 patients. In previous studies, primary sites were identified in 20 to 30% of patients with MUO, including lymphoma, sarcoma, and melanoma [17, 18]. These three malignancies should be considered while diagnosing patients with MUO or suspected CUP.

In a previous study, 6% of patients with suspected CUP were diagnosed with non-malignant disease, while 9% of them with non-neoplastic disease were found to have tuberculosis (2 patients in our study) [19]. Taking these findings into consideration, it is important to proceed with pathological examinations for patients with MUO or suspected CUP.

CUP patients with favorable risk often respond to treatment based on the putative primary site, and their survival may be prolonged. In our study, the median OS of 57 patients with favorable risk was 27 months. Despite heterogeneity, patients with unfavorable risk have been treated as a single entity, primarily with platinum-based combination chemotherapy. However, the prognosis of these patients remains to be poor [6].

Studies of site-specific therapies based on molecular profiles revealed survival improvement for CUP compared to those treated with empiric chemotherapies [20, 21]. Recently, two randomized trials enrolling patients with unfavorable-risk CUP into site-specific therapy by gene expression profile (GEP) or empiric regimen arms have failed to demonstrate statistically significant improvements in progression-free survival and OS by site-specific therapy [22, 23].

IHC can be performed in the pathology laboratories of all major hospitals, and the results can be obtained within a few days. IHC requires more stains (the median number of IHC stain used in our study was six), and the antibody selection for IHC examination relies mainly on pathologist’s experience. Previous studies indicate that GEP is a more appropriate for a diagnostic approach for predicting the primary site of CUP than conventional IHC [24–26]. However, GEP is an expensive and time-consuming method for routine clinical testing.

Taking into account the presumed CUP etiology [27–29], the poor efficacy of empiric chemotherapies for unfavorable-risk CUP [6], and previous studies on site-specific therapies [20, 21], we had attempted site-specific therapies for patients with unfavorable-risk CUP by IHC examinations [7].

In a previous study, we attempted to predict a primary site based on histopathology, IHC examinations, KRAS mutation, elevation of tumor markers (CA19-9 and CEA), and metastatic site distribution to distinguish gastric, pancreatic, and bile duct cancer profiles [7]. In this present study, we re-evaluated these three profiles, and it was difficult to distinguish them. For this reason, we categorized these as a single GPBC profile and reviewed clinical outcomes, which accounted for 41% of patients with predicted primary-sites. In addition, the prognosis of patients within GPBC profiles treated with systemic therapy was poor. In our study, the median OS of patients treated with site-specific therapy by the IHC panel was comparable to that of patients treated with empiric chemotherapies [6]. Furthermore, in our study, patients with UtCC and RCC profiles had longer median OS. CUP with profiles suggesting RCC and UtCC may be classified as one of the subtypes with a favorable risk [15, 16].

The major limitation in our study is that the IHC panel for CUP was not validated using metastatic tumors of known primary-sites. Despite the fact that the number of patients with UtCC and RCC profiles in this study was small, our findings indicate that prediction of primary site based on IHC examination and distribution of metastatic sites for unfavorable-risk CUP is effective for identifying subgroups responsive to site-specific therapy. Previous studies suggested that patients with unfavorable-risk CUP presumed to have therapy-responsive tumors had longer survival than those with less responsive tumors [20, 22, 30]. A recent phase II study of nivolumab for patients with unfavorable-risk CUP revealed a therapeutic benefit [31]. Furthermore, IHC examinations may aid in the selection of patients with unfavorable-risk CUP who are responsive to ICIs.

In conclusion, the regional cancer hospital has played an important role in the management of MUO and CUP, as this is supported by oncologists with expertise in CUP, pathologists, and interventional radiologists. It emphasizes the significance of early consultation or referral of MUO and CUP to a regional cancer hospital. The outcomes of unfavorable-risk CUP remain poor. Considering the presumed etiologies of CUP, prediction of primary sites on IHC examination and distribution of metastatic sites for CUP may be reasonable. However, the clinical benefit of site-specific therapy based on predicted primary sites for all patients with unfavorable-risk CUP has not been recommended in practice. The result of our study suggests that the IHC examination plays an important role in identifying subgroups that would benefit from site-specific therapy.

## Supplementary Information

Below is the link to the electronic supplementary material.

Supplementary file1 (PDF 181 KB)

## Data Availability

The authors confirm that the data supporting the findings of this study are availabe within the article and its supplementary materials.
