# Supplementary material for: Clinical outcomes of patients diagnosed with cancer of unknown primary or malignancy of undefined primary origin who were referred to a regional cancer center
Source: Int J Clin Oncol. 2023 Mar 10;28(5):644–53. doi: 10.1007/s10147-023-02316-y (PMC10119062; doi:10.1007/s10147-023-02316-y)

Appendix table 1 Patients' characteristics between predictable and non-predictable primary-site

	Predictable primary-site (n=114)	Non-predictable primary-site (n=41)	
Male / Female	60 / 54	29 / 12	
Age median (range)	66 (28 - 83)	64 (30 - 79)	
ECOG PS	1 (0 - 3)	1 (0 - 4)	
Number of metastatic sites	1 (1 - 5)	1 (1 - 4)	
	n(%)	n(%)	
Metastatic site			P value (Predictable vs Non-predictable)
Lymph node	62 (54)	25 (61)	0.582
Bone	31 (27)	19 (46)	0.0321
Liver	22 (19)	5 (12)	0.348
Lung	17 (15)	2 (5)	0.105
Peritoneum	35 (31)	4 (10)	0.0108
Histopathology			
Squamous cell carcinoma	11 (10)	12 (29)	0.004

Appendix table 2 Therapy for 206 patients with caner of unknown primary at the Aichi Cancer Center Hospital

	Favorable-risk subtypes (n=57)	Unfavorable-risk subtype (n=149)
Systemic therapy only	23	77
Surgery only	2	1
Radiotherapy only	1	6
Systemic therapy + Surgery	8	9
Systemic therapy + Radiotherapy	10	35
Systemic therapy + Surgery + Radiotherapy	10	12
Surgery + Radiotherapy	1	0
Best supportive care only	2	9

Appendix table 3 Regimens of the first-line systemic therapy for 133 patients with unfavorable-risk subgroup of cancer of unknown primary

Predicted primary-site by the algorithm	Non-predicted primary-site (n=30)	Breast / skin appendage / salivary gland cancer profile (n=13)	Non-small cell lung cancer profile (n=8)	Gastric / pancreatic / bile duct cancer profile (n=58)	Urinary tract cancer profile (n=6)	Renal cell cancer profile (n=4)	Ovarian / endometrial cancer profile (n=5)	Uterus cervical cancer profile (n=7)	Melanoma of unknown primary (n=2)
Regimens									
Platinum+Taxane	19	8	6	1	1		5	3	
CDDP+VP-16	1								
CBDCA+GEM	3			2	4				
CDDP+PEM			1						
Platinum+5-FU	4	1						3	
L-OHP+5-FU	1	1		51	1				
GEM+5-FU	1			1					
Anthra/Taxane		1							
Single agent†	1			2					
Immuno check point inhibitor††			1	1				1	2
Endocrine‡		2							
VEGFR TKI§						4			
Time to treatment failure months (95% C.I.)	4.0 (2 - 6)	11.0 (0 - 19)	4.0 (0 - 6)	3.5 (2 - 4)	2.0 (0 - NA)	24.0 (18 - NA)	10.0 (0 - NA)	9.0 (1 - NA)	3 .0 and 4.0

Anthra, anthracycline, CBDCA, carboplatin, CDDP, cisplatin, 5-FU, 5-fluorouracil, GEM, gemcitabine, L-OHP, oxaliplatin, NA, not available, Platinum, cisplatin or carboplatin, VEGFR TKI, vascular endothelial growth factor receptor tyrosine kinase inhibitor

†S-1 or docetaxel, ††nivolumab, pembrolizumab, or combination with ipilimumab and nivorumab, ‡axitinib, sunitinib, pazopanib, §anastrozole, letrozole

Appendix table 4 Subgroup analysis for overall survival of unfavorable-risk subgroup treated with systemic therapy

n=133	Univariated analysis					Multivariated analysis		
Factors		Number of patients	Hazard ratio	95% C.I.	P value	Hazard ratio	95% C.I.	P value
Age	65> / 65=<	61 / 72	0.99	0.66 - 1.48	0.97			
Sex	Male / Female	78 / 55	1.23	0.21 - 0.99	0.32			
ECOG PS	0, 1 / 2, 3	95 / 38	1.94	1.27 - 2.95	0.002	1.9	1.24 - 2.90	0.003
Number of metastatic sites	1, 2 / 3=<	107 / 26	2.8	1.75 - 4.48	0.00002	1.92	1.06 - 3.48	0.03
Liver metastasis	No / Yes	106/27	2.84	1.77 - 4.55	0.00002	1.9	1.05 - 3.46	0.04
Bone metastasis	No / Yes	92 / 41	1.44	0.94 - 2.21	0.09			
Predictable primary-site	Predictable / Non-predictable	103 / 30	1.22	0.75 - 1.98	0.42			

PS, performance status

Appendix figure

Final diagnosis at the Aichi Cancer Center Hospital	Events	Median OS months	95% C.I.
MUO	10 / 12	0.75	0 – 3
Provisional CUP	9 / 13	1	1 – 12
Confirmed CUP	134 / 206	16	13 – 20
Primary-site identified	16 / 38	24	6 - NE

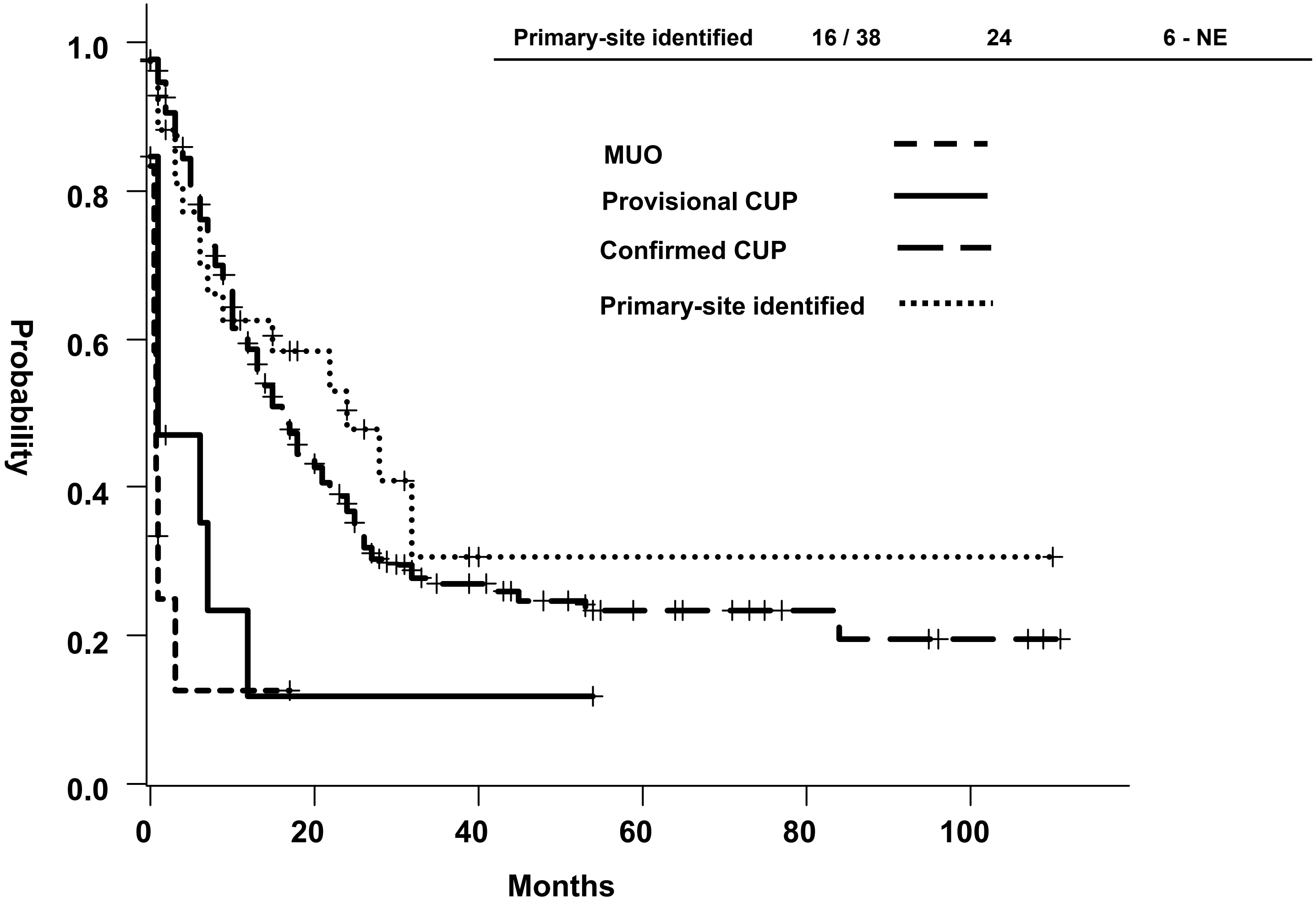

Supplement: Supplementary file 1 — Supplementary file1 (PDF 181 KB) [file 10147_2023_2316_MOESM1_ESM.pdf]
